# Room‐Temperature Quantum Memories Based on Molecular Electron Spin Ensembles

**DOI:** 10.1002/adma.202101673

**Published:** 2021-06-09

**Authors:** Samuel Lenz, Dennis König, David Hunger, Joris van Slageren

**Affiliations:** ^1^ Institute of Physical Chemistry and Center for Integrated Quantum Science and Technology University of Stuttgart Pfaffenwaldring 55 D‐70569 Stuttgart Germany

**Keywords:** microwave pulse storage, molecular quantum bits, organic radicals, quantum memories, quantum technologies

## Abstract

Whilst quantum computing has recently taken great leaps ahead, the development of quantum memories has decidedly lagged behind. Quantum memories are essential devices in the quantum technology palette and are needed for intermediate storage of quantum bit states and as quantum repeaters in long‐distance quantum communication. Current quantum memories operate at cryogenic, mostly sub‐Kelvin temperatures and require extensive and costly peripheral hardware. It is demonstrated that ensembles of weakly coupled molecular spins show long coherence times and can be used to store microwave pulses of arbitrary phase. These studies exploit strong coupling of the spin ensemble to special 3D microwave resonators. Most importantly, these systems operate at room temperature.

## Graphical abstract

Ensembles of weakly exchange coupled organic radicals form strongly coupled systems with 3D microwave resonators. Time‐domain pulsed microwave investigations reveal long, largely temperature‐independent quantum coherence times. The room‐temperature storage and retrieval of microwave pulses is demonstrated.

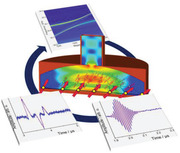

## 1 Introduction

Quantum technologies rely on the ability to perform coherent manipulations of systems that possess two or more levels. Such systems are called quantum bits, or qubits for short. Many platforms for the implementation of quantum bits have been proposed.^[^ ^1^ ^]^ For practical quantum operations, it appears that qubits based on superconducting circuits are ahead of the pack because of their speed of operation and (limited) scalability.^[^ ^2^ ^]^ However, superconducting circuits must be cooled down to millikelvin temperatures because their coherence times are short (typically several microseconds).^[^ ^3^ ^]^ Because in a functional quantum device, operations will be carried out on many qubits that must be synchronized, there is a need for units to store quantum information for longer times. Such units are called quantum memories.^[^ ^4^ ^]^ Furthermore, a means must be devised to transfer quantum information between quantum memory and quantum bit. For transfer of quantum information between quantum processors and memories, photons are most suitable.^[^ ^5^ ^]^ Superconducting quantum bits are addressed by means of microwave radiation and quantum memories in this context thus need to be able to store microwave photon states. To this end, resonant structures for electromagnetic radiation can be used to strongly couple quantum bits and quantum memories to quantized cavity modes of the electromagnetic field. The strong coupling generates a hybrid quantum system that allows mapping the qubit state onto the cavity field state.^[^ ^6^ ^]^ This strategy has given rise to the field of cavity quantum electrodynamics and has already been used to couple two superconducting qubits together via a cavity bus.^[^ ^7^ ^]^ A second application of quantum memories is in quantum repeaters for quantum communication. Because telecom wavelengths are in the near‐infrared, for this application, optical quantum memories are required that store optical photon states.^[^ ^8^ ^]^

Considering the choice of material platform for designing microwave quantum memories, electron spins in solids can be easily addressed by microwave pulses and have been shown to possess excellent coherence times up to seconds, for some systems even up to room temperature.^[^ ^9^, ^10^ ^]^ Unfortunately, the coupling of a single electron spin to a photon is very weak. Although resonant structures can be tailored to improve single‐spin coupling,^[^ ^11^, ^12^, ^13^ ^]^ the weakness of the coupling renders addressing individual spins by means of coupling to cavity modes very challenging. This can be overcome by using an electron spin ensemble rather than single spins, because the coupling strength of a spin ensemble to an electromagnetic resonator mode is proportional to the square root of the number of electron spins in the ensemble.^[^ ^14^ ^]^

**Figure 1:**
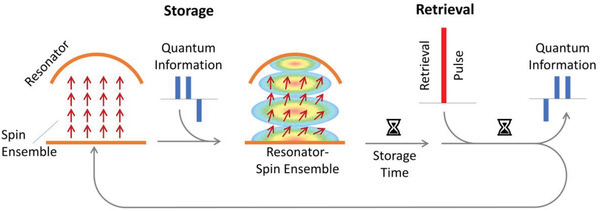
Schematic overview of the idea of a quantum memory, in which microwave pulses are stored in a spin ensemble. First an arbitrary sequence of microwave pulsed is stored in a spin ensemble that is strongly coupled to a 3D microwave resonator. After the desired storage time, the microwave pulse sequence is recalled by a strong retrieval pulse.

This then leads to the general idea of the implementation of a microwave quantum memory using spin ensembles and microwave resonators (**Figure** **1**). The state to be stored is encoded in a weak microwave pulse and sent to the hybrid quantum system constituted of an electron spin ensemble that is strongly coupled to a microwave resonator, where it is stored. When the quantum state is needed again, a strong microwave pulse is sent to the resonator, which leads to deterministic retrieval of the quantum state that is emitted as a microwave photon to be used as desired.

Strong coupling between cavity and electron spin ensembles has been observed in a variety of spin systems, including organic radicals,^[^ ^15^, ^16^, ^17^, ^18^, ^19^, ^20^ ^]^ phosphorous dopants in silicon,^[^ ^21^, ^22^ ^]^ P1 and nitrogen vacancy (NV) defects in diamond,^[^ ^23^, ^24^, ^25^, ^26^, ^27^, ^28^, ^29^, ^30^, ^31^, ^32^ ^]^ N@C_60_,^[^ ^33^ ^]^ rare earth dopants in oxides,^[^ ^34^, ^35^ ^]^ transition metal complexes,^[^ ^36^, ^37^ ^]^ and molecular nanomagnets.^[^ ^38^ ^]^ Collective coupling strengths of up to 200 MHz,^[^ ^38^ ^]^ and cooperativities up to 4300 have been reported,^[^ ^17^ ^]^ and strong coupling has been observed at temperatures up to room temperature.^[^ ^15^, ^18^, ^19^, ^20^ ^]^ In addition, sizable effort has been directed towards the investigation on the strong coupling of magnetically ordered systems with magnon excitations to resonators.^[^ ^39^, ^40^, ^41^ ^]^

In contrast, the quantum dynamics of strongly coupled spin ensembles in the time domain has been much less investigated. This is surprising, since strongly coupled spin ensembles are only useful if they can actually be used as quantum memories. In early experiments, Fourier transformation of the cavity ringing after a microwave pulse gave the frequencies of the two polariton modes.^[^ ^19^ ^]^ In a similar measurement at 40 mK, it was concluded that the strong coupling protects the system from loss of phase memory due to inhomogeneous distribution of spin resonance frequencies.^[^ ^30^ ^]^ This is called the cavity protection effect. It was shown that this effect can be strengthened by judiciously chosen pulse schemes.^[^ ^42^ ^]^ By spectral hole burning, long lived dark states are created that have dephasing times of up to 26.7 µs. A very elegant, quasi‐non‐destructive measurement scheme measures the evolution of the cavity resonance frequency in time, allowing continuous monitoring of the magnetization of the sample.^[^ ^31^, ^43^ ^]^ Furthermore, it has proved possible to store microwave pulses in strongly coupled NV spin ensembles.^[^ ^32^ ^]^ The dynamics of collective states was also studied by strong microwave pulses, recording the free induction decay in an experiment more akin to conventional pulsed magnetic resonance, featuring much stronger excitations.^[^ ^22^ ^]^ Self refocusing echoes were observed in inhomogeneously broadened spin systems.^[^ ^44^, ^45^ ^]^ Finally, microwave pulse storage and retrieval experiments were carried out in superconducting resonators at temperatures up to 2 K.^[^ ^46^ ^]^ However, these measurements were all carried out at low to ultralow temperatures.

Here, we present investigations of ensembles of stable organic radicals (benzene complex of α,γ‐bisdiphenylene‐β‐phenylallyl (≡ BDPA·Bz), Figure S1, Supporting Information), by means of a specially designed 3D resonator of the Fabry–Pérot type that has highly enhanced microwave field homogeneity compared to conventional electron paramagnetic resonance (EPR) resonators.^[^ ^47^ ^]^ We achieve the strong coupling regime up to room temperature. We investigate the spin dynamics by pulsed microwave measurements with both weak and strong pulses. We demonstrate that we can store and faithfully retrieve microwave magnetic field pulses with arbitrary phase, even at room temperature. Furthermore, we show that this is only possible because the spin ensemble consists of two sub‐ensembles, one which experiences nearest‐neighbor exchange coupling in a spin chain, and one in which there are essentially no exchange couplings. These results show the way to engineer such hybrid spin ensembles to increase the efficiency and fidelity of quantum information storage.

## 2 Theory

The conventional description of the interaction between a (microwave) magnetic field and an electron spin, leading to the magnetic resonance phenomenon neglects the quantum mechanical nature of the electromagnetic field. However, if the number of photons is no longer very large, the microwave field in the cavity must be treated quantum mechanically, leading to the Jaynes–Cummings model, with the Hamiltonian:^[^ ^48^ ^]^

(1) $$\begin{array}{c} \begin{array}{l} H=H_{\text{cavity}}+H_{\text{spin}}+H_{\text{interaction}}\\ \;\;\;\;=\hbar \omega_{c}a^{†}a+\hbar \omega_{s}{\hat{S}}_{z}+i\hbar g_{s}\left({\hat{S}}_{+}a-{\hat{S}}_{-}a^{†}\right) \end{array} \end{array}$$

Here ω_c_ is the resonance frequency of the cavity, ω_s_ is the resonance frequency of each spin and *g*_s_ is the single spin–photon coupling strength. The operators *a* ^†^ and *a* create and annihilate photons in the cavity. Here $H_{\text{spin}}$ is assumed to consist only of the Zeeman interaction, and that the spin–photon interaction $g_{s}|B_{\text{photon}}|(g\mu_{B}/2\hbar )=\sqrt{\hbar \omega \mu_{0}/2V_{\mathrm{mode}}}(g\mu_{B}/2\hbar )\approx 10^{‐1}\text{Hz}$ is much weaker than cavity and spin resonance frequencies (rotating wave approximation). The eigenstates are described in terms of the number of photons in the cavity (Fock state |*n*〉) and the spin orientation (|↓〉, or |↑〉, assuming *S*  =  ½). The ground state of the system is then |0↓〉. At zero detuning Δ  =  ω_s_ – ω_c_  =  0, and *n*  =  0, the first two excited states are $\left|\;\;\pm \;\rangle \;=\;\frac{1}{\sqrt{2}}(|0↑\rangle \;\pm \;\;|1↓\rangle \right)$.^[^ ^49^ ^]^ This means that the excitation is equally shared between cavity photon number and spin; the corresponding quasi‐particle is called polariton. Conversely, if the system is prepared in a state of pure spin or cavity excitation, the excitation energy will oscillate between spin and cavity at a frequency that depends on the spin–photon coupling strength (vacuum Rabi oscillations). This oscillation is damped by rates given by the loss of energy from the cavity (cavity dissipation) and decoherence of the spin. Both are usually many orders of magnitude larger than *g*_s_.

This changes if an ensemble of spins is considered. For each spin in the ensemble that is excited, an equal amount of energy is added to the system, suggesting a harmonic‐oscillator‐like energy spectrum, which allows the description of the spin system by bosonic operators (the so‐called Holstein–Primakoff transformation).^[^ ^50^ ^]^ If we consider that the excitation energy is shared by all the spins, the excitation becomes spin‐wave‐like in nature, with the magnon as the corresponding quasi‐particle. The spin system is then considered a giant spin, for which the total spin is *S*_T_  =  *N*/2 (assuming *T* << *ħω*_s_). If the number of photons and magnons is small compared to the number of spins, the hybrid spin‐cavity system can be described as two interacting harmonic oscillators:

(2) $$\begin{array}{c} H=\hbar \omega_{c}a^{†}a+\hbar \omega_{c}b^{†}b+i\hbar \Omega_{\text{eff}}\left(ab^{†}-a^{†}b\right) \end{array}$$

with b†=N−12∑i=1NS^−,i,b=N−12∑i=1NS^+,i. In that case, the polariton modes have energies given by:

(3) E±=12ℏωc+ωs±Δ2+4Ωeff2

The crucially important result of this description is that the effective collective coupling spin–photon strength is given by $\Omega_{\text{eff}}=\sqrt{N}g_{s}$, that is, the coupling is increased by a factor $\sqrt{N}$. This means that by using large enough numbers of spins the cavity‐spin coupling can be made larger than the spin and cavity dissipation frequencies. Here, it has been assumed that all spins have the same excitation frequency, which, in real life, is not the case. We will consider the effects of such inhomogeneous broadening below, when we discuss the probing of hybrid spin‐cavity systems.

The steady‐state response of the hybrid system can be studied by sending weak microwave radiation to the cavity and recording the radiation returning, as a function of microwave frequency ω_p_ and external magnetic field *B*_0_, for example by employing a network analyzer. To take into account the inhomogeneous broadening of the ensemble, we divide the spin ensemble into sub‐ensembles (spin packets) that are each assumed to have the same excitation frequency. The scattering parameter *S*_11_, that is, the ratio between input and output fields, is then given by:

(4) $$\begin{array}{c} S_{11}=\frac{B_{\text{out}}}{B_{\text{in}}}=1-\frac{2\kappa_{e}}{i\left(\omega_{c}-\omega \right)+\left(\kappa_{e}+\kappa_{i}\right)+\sum_{j=1}^{N}\frac{-2N_{j}g_{s}^{2}\left\langle {\hat{S}}_{z,j}\right\rangle }{i\left(\omega_{s,j}-\omega \right)+\gamma_{j}}} \end{array}$$

Here κ_e_ is the external dissipation rate, that is, the losses due to the coupling of the resonator to an external microwave circuit (e.g., source, detector,…), κ_i_ is the internal dissipation rate (the losses inside the cavity, essentially due to the finite resistance of the cavity wall) and γ the spin dissipation rate (spin–spin and spin–lattice relaxation). N is the number of spin packets and *N*_*j*_ is the number of spins in each spin packet. The expression contains the *Ŝ*_z_ expectation value to take into account the effects of finite temperature and interactions within the sample.^[^ ^20^ ^]^ In this case the collective spin–photon coupling strength is given by $\Omega_{\text{eff}}=\sqrt{-2N_{j}\left\langle {\hat{S}}_{z}\right\rangle }g_{s}$. In the *S*_11_(*B*_0_,ω_p_) diagram, this leads to an anticrossing of the absorption lines describing cavity and spin excitations in the field–frequency diagram. The observation of such an anticrossing, where the splitting is larger than the line widths of cavity and spin resonance lines, is considered a signature of strong coupling between cavity and spin ensemble. In terms of rates, this means that the coupling rate Ω_eff_ must be much higher than the cavity loss rate κ and the spin decoherence rate γ. The cooperativity parameter *C*, defined as $C=\Omega_{\text{eff}}^{2}/\left(\kappa \gamma \right)$, with *C* >> 1, summarizes this requirement.

The time‐dependent response can be calculated by numerically solving a closed set of differential equations describing the rates of change of the photon number and spin excitations.^[^ ^51^ ^]^ To this end, a driving term describing the effect of microwave irradiation must be added to the Tavis–Cummings Hamiltonian (which is the Jaynes–Cummings Hamiltonian, Equation (1), for many spins). Using a coordinate frame that rotates with the precession frequency of the spins at the center of their distribution, this Hamiltonian reads:

(5) $$\begin{array}{l} H=\hbar \Delta_{\text{cs}}a^{†}a+\overset{N}{\underset{i}{\sum }}\hbar \Delta_{s,i}{\hat{S}}_{z,i}+\overset{N}{\underset{i}{\sum }}i\hbar g_{s,i}\left({\hat{S}}_{+,i}a-{\hat{S}}_{-,i}a^{†}\right)+\\ \;\;\;\;\;\;\;i\hbar \sqrt{2\kappa_{e}}\left({B^{\prime }}_{\text{in}}\left(t\right)a^{†}-B_{\text{in}}^{\prime *}\left(t\right)a\right) \end{array}$$

Here Δ_cs_  =  ω_c_ – ω_s,c_ is the detuning between the cavity resonance and the spin resonance at the center of the inhomogeneous distribution, and Δ_s,i_  =  ω_s,i_ – ω_s,c_ is the difference between spin resonance frequencies of spin *i* and that at the center of the distribution. The probing field is denoted *B*’ because in the rotating coordinate frame, it corresponds to the down‐converted (in signal processing terms) field, i.e., *B*’ = *B*exp(–*iω*_s,c_ *t*). The resulting differential equations that can be solved numerically are given in refs. ^[^ ^51^, ^52^ ^]^ and not repeated here.

## 3 Results and Discussion

### 3.1 Investigation of the Steady‐State Properties by Continuous Wave Measurements

**Figure 2:**
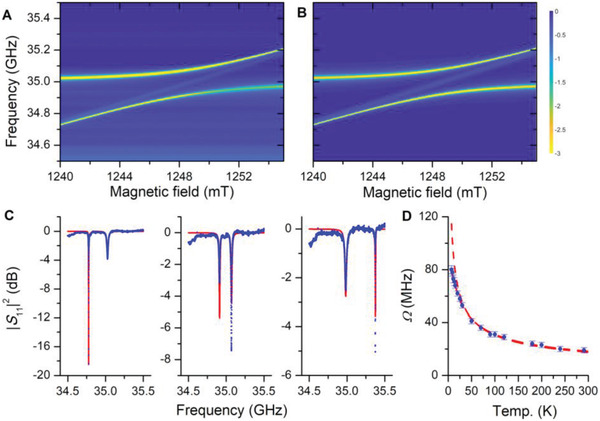
Steady‐state probing results. Measurements were carried out on a 9.4 mg, 5 mm pressed pellet of BDPA·Bz using the first mode in a copper Fabry–Pérot resonator at 7 K. A) Reflected microwave intensity |*S*_11_|^2^ as a function of magnetic field and microwave frequency, and B) corresponding simulation. C) Cuts along the frequency axis at magnetic fields of 1241.69 mT (left), 1248.00 mT (middle) and 1261.33 mT (right). Symbols are experimental data; red lines are simulations (see text). D) Effective coupling spin–photon coupling constant as a function of temperature (symbols) and corresponding fit (dashed line).

First we investigated the steady‐state response of the hybrid quantum system consisting of a 9.4 mg (≈6 × 10^18^ spins) pressed powder sample of the stable free radical α,γ‐bisdiphenylene‐β‐phenylallyl benzene solvate (, abbreviated BDPA hereafter) mounted on the bottom, flat mirror of a semi‐confocal copper Fabry–Pérot resonator (FPR).^[^ ^47^ ^]^ To this end, we recorded the radiation reflected from the cavity (*S*_11_ scattering parameter) as a function of external magnetic field *B*_0_ and microwave frequency by means of a vector network analyzer at a temperature of *T*  =  7 K (**Figure** **2**A). The first resonance mode (i.e., that with smallest inter‐mirror separation) of the cavity was tuned to 35.000 GHz prior to the measurement. The measurement result displays a clear anti‐crossing between a field‐independent feature at 35.000 GHz and a linearly field‐dependent feature. The former feature belongs to the cavity resonance, while the latter is due to the magnetic resonance excitation of the electron spins. The anti‐crossing gap size is clearly much larger than the width of both excitations, qualitatively demonstrating that the system is in the strong coupling regime. Fitting the data to Equation (4) gave the following fit parameter values: decoherence rate γ/2π =  1.5(5) MHz, internal cavity dissipation κ_i_/2π  =  2.0(5) MHz, external cavity dissipation κ_e_/2π  =  13.0(5) MHz and the effective collective spin–photon coupling strength Ω_eff_/2π  =  80(3) MHz. Because Ω_eff_ >> *γ, κ*, the system is, in fact, in the strong coupling regime. In fact, the cooperativity *C*  =  2.9(9) × 10^2^, which is the largest reported value for simple paramagnetic systems. At first sight, it is remarkable that the spin excitation width is only 1.5 MHz, where one would expect a sizable distribution in intermolecular magnetic dipolar interactions exceeding this value. In fact, the dipolar interaction, calculated in the point dipole approximation on the basis of the crystal structure of BDPA^[^ ^53^ ^]^ amounts to 50 MHz. However, many organic radicals, including BDPA,^[^ ^54^ ^]^ have exchange narrowed resonance lines due to non‐negligible intermolecular exchange interactions, and, as a consequence, the sample can be considered a spin ensemble of identical spins with equal resonance frequencies ω_s_ and relaxation rates γ. The collective coupling strength, $\Omega_{\text{eff}}=\sqrt{-2N\left\langle {\hat{S}}_{z}\right\rangle }g_{s}$, depends on the number of spins (*N*  =  1.1 × 10^19^), their polarization <*Ŝ*_z_>, and the single‐spin–photon coupling strength *g*_s_. The last was estimated to be *g*_s_/2π  =  0.139 Hz for the used resonator mode (see Table S2, Figure S2, Supporting Information). The collective coupling strength decreases with increasing temperature (Figure 2D, Table S1, Supporting Information). In view of $\Omega_{\text{eff}}=\sqrt{-2N\left\langle {\hat{S}}_{z}\right\rangle }g_{s}$, this is expected, because the spin polarization <*Ŝ*_z_> depends on temperature. The expected <*Ŝ*_z_> for an ensemble of non‐interacting spins‐½ is given by <*Ŝ*_z_>(*T*)  =  –½tanh(*ħω*_s_/*kT*). At high temperatures, the result matches the experimental Ω_eff_ perfectly (Figure 2D), but deviations are observed at low temperatures, where Ω_eff_ is smaller than calculated. These deviations are attributed to exchange interactions between the BDPA molecules, and indeed BDPA has been reported to behave as a 1D Heisenberg antiferromagnet.^[^ ^55^ ^]^ In fact, a clear agreement between collective coupling strengths and magnetic susceptibility measurements was found for exchange coupled radical dimers, recently.^[^ ^20^ ^]^ Hence, SQUID magnetometric measurements were carried out (see Figure S3, Supporting Information) from which the exchange coupling constant was determined to be *J*/*k*_B_ = −4.9 K, in agreement with previously reported values.^[^ ^56^, ^57^ ^]^ Note that these data indicate the presence of a small, uncoupled impurity (see below). The temperature‐dependent *Ŝ_z_* expectation value, <*Ŝ_z_* > (*T*), can be calculated from the density matrix as ${\hat{S}}_{z}(T)=\frac{1}{N_{c}}\text{tr}\left[\hat{\rho }(T)\overset{N}{\underset{i=1}{\sum }}\left\langle {\hat{S}}_{z,i}\right\rangle \right]$, with $N_{c}$ the chain length and the density matrix operator defined as $\hat{\rho }(T)=\exp(-H_{\text{spin}}/kT)\text{/tr}(\exp(-H_{\text{spin}}/kT))$. This was carried out for a chain length of $N_{c}$  =  12, assumed to be in the long‐chain limit. The resulting <*Ŝ_z_* > (*T*) were used to obtain a very good fit of the data in Figure 2D. From this fit, we obtained a single‐spin photon coupling constant of *g*_s_/2π  =  0.145(6) Hz, in perfect agreement with that obtained from microwave simulations (Section S3, Supporting Information).

Inspecting the experimental data in Figure 2A more carefully reveals a faint line inside the anticrossing. Such a line has been observed before and has been attributed to states that formally have no cavity part and thus do not couple to electromagnetic radiation (dark states).^[^ ^58^, ^59^ ^]^ In the giant spin picture, they correspond to excitations that change the total spin,^[^ ^60^ ^]^ which are EPR‐forbidden. Due to inhomogeneous broadening, these excitations gain some allowedness and can be observed. In BDPA, the inhomogeneous broadening is due to *g*‐value anisotropy, and becomes noticeable in CW EPR spectra below 12 K.^[^ ^57^ ^]^ To model the inhomogeneous distribution and simulate the plot in Figure 2A, we have discretized the ensemble into a large number of smaller ensembles (spin packets), each with a different central resonance frequency, where the number of spins in each spin packet is given by the spectral distribution function, here assumed *q*‐Gaussian (see Supporting Information for more details). The fits (Figure 2B,C) very faithfully reproduce the experiment, including the faint central line and reveal an inhomogeneous full‐width‐at‐half‐maximum linewidth of γ_inh_  =  11.9(3) MHz. The minor discrepancies between simulated and experimental intensities may be due to the frequency dependence of the coupling efficiency of the radiation into the cavity, as well as the coax‐to‐waveguide transition which is not taken into account in the simulations. Interestingly, the line width of the polariton modes Γ  =  ½(κ + γ_hom_) and the effective collective coupling constant Ω_eff_ are not affected by the width of the spectral distribution, as long as Ω_eff_ >> γ_inh_. As a consequence, the strong coupling between spin ensemble and the resonator effectively removes the contribution of inhomogeneities to spin dephasing, which has been called the cavity protection effect.^[^ ^42^, ^58^, ^59^ ^]^

### 3.2 Investigation of the Quantum Dynamics by Pulsed Measurements

To investigate the cavity protection effect in the time domain, we have used weakly perturbing microwave driving pulses, and these results are discussed first. Second, we performed the canonical pulsed EPR measurement, that is, the Hahn echo measurement on the strongly coupled hybrid system. Finally, we assess the performance of these systems as quantum memories.

**Figure 3:**
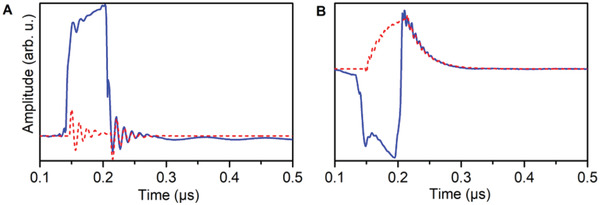
Weak pulse measurements. Reflected microwave amplitude (in‐phase component, blue) recorded on a 9.4 mg BDPA.Bz pressed powder pellet at 7 K and 1248.8 mT in the time domain. The system was excited by a 65 ns rectangular pulse with a pulse power of 5 µW. Red dashed lines are simulations that only take into account radiation emitted by the cavity. In the experiment, the radiation reflected at the coupling hole is also detected. A) Excitation occurred at zero detuning between cavity and spin mode (ω_p_  =  ω_c_  =  ω_s_  =  35.000 × 2π GHz), B) excitation occurred at the lower polariton mode (ω_p_  =  34.922 GHz).

For the first experiment, the previously used BDPA sample in the copper FPR was connected to the pulsed Q‐band bridge and cooled to 7 K, with the FPR tuned to 35.000 GHz. **Figure** **3**A displays the microwave intensity reflected from the Fabry–Pérot resonator upon irradiation with a weak, 65 ns, 5 µW rectangular probe pulse at ω_p_/2π = 35.000 GHz and an external field of *B*_0_  =  1248.8 mT (i.e., in the middle of the anticrossing gap in the S_11_ field/frequency plot). This is a weak pulse (six orders of magnitude less than the maximum output power of the bridge), and corresponds to inserting about 2 × 10^9^ photons into the resonator. Due to the homodyne nature of the bridge, the signal is detected in the time domain after down conversion to a baseband signal. The overall rectangular shape of the signal corresponds to the microwave intensity reflected off the waveguide/resonator transition due to imperfect matching (no coupling iris is implemented). The sign of this contribution to the detected intensity depends on the relative phase of the reflected intensity and the reference arm and can thus be positive and negative. Superimposed on the rectangular pulse, as well as after the pulse, clear damped oscillations with identical frequencies of 78 MHz are observed. We attribute these to vacuum Rabi oscillations:^[^ ^19^, ^22^ ^]^ in the Jaynes–Cummings picture, at zero detuning (cavity and spin resonance frequencies are the same), the two polariton mode eigenstates are superpositions between cavity and spin excitations (see Section 2). Probing the system with a pulse with frequency ω_p_  =  ω_c_  =  ω_s_ adds photons into the system, exciting superpositions of the polariton eigenstates $\left|1↓\rangle \;=\;\frac{1}{\sqrt{2}}(|\;+\;\rangle \;+\;\;|-\rangle \right)$, which themselves are not eigenstates of the system. As a consequence, in a time‐dependent picture, in a coordinate frame rotating with frequency ω_p_ (as in our homodyne detection system), the system will start to oscillate between cavity and spin states according to cos(Ω_eff_ *t*)|1↓〉 – *i*sin(Ω_eff_ *t*)|0↑〉, leading to vacuum Rabi oscillations. Only when the excitation is in the cavity state, radiation can exit the cavity and reach the detector, resulting in an oscillatory behavior of the free‐induction decay (FID)‐like detector signal. The oscillation frequency (78 MHz) should therefore correspond to the effective collective coupling strength, and indeed it does (80 MHz, see above and Table S1, Supporting Information). The oscillations decay mono‐exponentially with a time constant of about 20 ns. The part of the signal due to the cavity‐spin ensemble system can be simulated according to the Hamiltonian in Equation (4), and the red, dotted lines in Figure 3A agree perfectly with the experimental results. The theoretical loss rate Γ  =  ½(κ + γ) ≈ ½κ is essentially determined by the cavity loss rate (κ/2π  =  15 MHz, corresponding to a time constant of 21 ns) which is much higher than the spin dephasing (γ/2π  =  1.5 MHz). Similar oscillations have been observed at millikelvin temperatures using diamond NV ensembles.^[^ ^42^ ^]^ Figure 3B displays the results from an analogous measurement using a slightly lower microwave frequency of 34.922 GHz corresponding to the lower polariton mode frequency. Two components can now be observed, one monoexponential decay superimposed on which is an oscillatory contribution with a frequency of 156 MHz. The latter corresponds to the energy gap between the two polariton modes, that is, twice the effective collective coupling rate. We attribute the non‐oscillatory component to the bottom polariton mode, and the oscillatory component to the top polariton mode. In this case the reflected radiation amplitude during the pulse is negative, which is due to amplitude signal having a different phase (our spectrometer does not feature quadrature detection), but this has no physical significance. The remaining questions are then why the oscillations decay during the pulse and, in fact, why the polariton modes are excited at all when employing nominal excitation frequencies far away from the polariton mode eigenfrequencies. After all, a 65 ns rectangular pulse roughly has a bandwidth of 15 MHz, that is, much less than the detuning between carrier and polariton frequencies as a simple fast Fourier‐Transform of a rectangular pulse shows (Figure S4, Supporting Information). However, Fourier transformation is not the intuitive way to analyze a non‐periodic signal such as a rectangular microwave pulse. An alternative method for time‐frequency analysis of such signals is provided by the continuous wavelet transform method. This method provides a means to visualize how the frequency content of a signal changes over time. Carrying out such an analysis on a 65 ns rectangular pulse (Figure S5, Supporting Information) shows that the frequency bandwidth of the rising and falling pulse edges is enormous, and can easily excite modes that are far away from the nominal microwave frequency. The preceding measurements underlined the possibility to study the hybrid cavity–spin ensemble system in the time domain. In the following, we study the dynamics using two‐pulse sequences that are commonly used in EPR.

**Figure 4:**
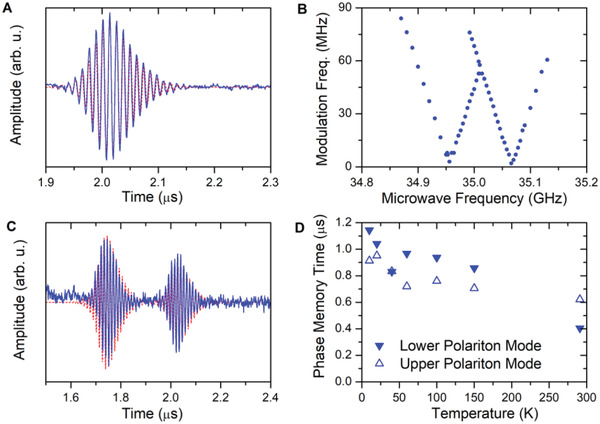
Spin echo measurement results. A) echo detected at 7 K after a pulse sequence consisting of two 25 ns pulses with a pulse power of 5 W, an interpulse delay of 1 µs and a frequency of ω_p_  =  ω_c_  =  ω_s_  =  2π × 35.000 GHz (zero detuning). Data were recorded on a 9.4 mg BDPA pellet at 1248.8 mT. B) Echo modulation frequency obtained by Fourier transformation of the echo signals obtained on a 21.5 mg BDPA pellet at 7 K after two 10 ns, 5 W microwave pulsed with a 1 µs interpulse delay as a function of microwave frequency. C) Double echo recorded on a 9.4 mg BDPA pellet at 1248.8 mT using a pulse sequence consisting of one 280 ns pulse and one 25 ns pulse separated by an interpulse delay of 1 µs. Both pulses had a pulse power of 5 W and a frequency of 35.000 GHz. D) Phase memory times derived from monoexponential fits of the echo decay recorded after excitation of upper and lower polariton modes at different temperatures.

We carried out echo measurements by exciting the system by means of two short, high‐power microwave pulses with variable interpulse delays. Such measurements are used in conventional pulsed EPR to refocus the dephasing of the spin ensemble that is due to its inhomogeneous resonance frequency distribution.^[^ ^61^ ^]^ The spin echo obtained after two 25 ns, 5 W pulses (corresponding inserting to ≈2 × 10^15^ photons into the cavity) with a 1 µs interpulse delay displays a pronounced modulation (**Figure** **4**A). The excitation frequency was chosen such that it lies in the middle of the two polariton mode frequencies (ω_p_  ω_c_  =  ω_s_). A Fourier transform of the echo signal reveals that the oscillation has a frequency of 78 MHz, which again corresponds to the effective collective coupling strength. This is corroborated by the fact that the oscillation frequency shows a linear dependence on the microwave frequency (Figure 4B, Figure S6, Supporting Information). At some frequencies, two distinct oscillations are observed, consistent with the excitation of both polariton modes. The echo intensity as a function of the interpulse delay (Figure S7, Supporting Information) decays mono‐exponentially with a time constant (phase memory time) of *T*_M_  =  1.4(1) µs, which corresponds to a decay rate of γ_M_/2π  =  0.114(8) MHz. Such a decay rate is much smaller than any decay rate (spin, cavity) observed so far. In fact, it is much longer than the phase memory time of BDPA determined to be of the order of 100 ns between 77 K and room temperature.^[^ ^62^, ^63^ ^]^ Spin echoes have been observed (at millikelvin temperatures) under conditions where the single‐spin–cavity coupling is large, but in most cases the cooperativity parameter *C* was small, meaning that the response of individual spins was measured.^[^ ^52^, ^64^, ^65^ ^]^ Therefore, that scenario is not applicable to the present case. We believe our findings to be consistent with the following scenario: i) The initial pulse creates coherence on both polariton modes. Although the polariton frequency is beyond the bandwidth of the pulse, we have seen that the instantaneous bandwidth of the pulse edges is sufficient to excite the polariton modes. The fact that coherence is generated by the pulse edges is corroborated by an experiment where an initial pulse of a much longer duration of 280 ns is employed. Here two echoes are observed after the second pulse, spaced by exactly 280 ns (Figure 4C), proving that both rising and falling edges of the first pulse generate coherences. ii) the generated coherence is transferred via interaction with the cavity to dark modes. These dark modes are weakly coupled to the cavity and thus do not decay efficiently via Purcell‐like photon emission. Importantly, however, these dark modes are outside of the spectral density of the spin ensemble. The (exchange‐narrowed) width of the spectral density distribution (γ_inh_  =  11.9 MHz, see above) is much smaller than the frequency difference (given by Ω_eff_ *=  *80 MHz, see above) between the ensemble center frequency and the polariton frequency. In fact, the echo signal could not be reproduced by using the model outlined in the theory section. This brings us back to the ≈10% impurity observed in the magnetic, as well as in the steady‐state microwave measurements. This second spin ensemble is not exchange narrowed and should thus have a much broader spectral distribution, and have substantial spectral density at the frequency of the polariton modes. iii) After being transferred to the impurity spin ensemble, the coherence dephases rapidly due to the inhomogeneity in the spectral distribution. iv) However, this type of dephasing can be refocused by a second microwave pulse, which is the basis of the spin echo measurement. Indeed, the magnetization is refocussed after a delay time equal to the interpulse separation. v) At the point where the magnetization is refocussed, interaction with the cavity radiation field is again possible, which excites the polariton modes and leads to a modulated echo signal. This scenario is somewhat similar to that proposed by Putz et al.^[^ ^29^, ^60^ ^]^ with two main differences: First, in their spin ensemble, the inhomogeneous distribution width exceeds the effective collective spin–cavity coupling. Second, in their experiments they inserted up to 10^4^ photons per spin into the cavity, whereas in our experiments we have about 10^4^ spins per photon. As a result, no significant hole burning is taking place in our experiments. Simulations of the system response, including a second spin ensemble that is associated with the impurity spins lead to a very satisfactory reproduction of the experimental data (Figure 4A,C), which corroborates the validity of our scenario. Finally, we have studied the temperature dependence of the phase memory time. For convenience, that is, avoiding the echo modulation, we directly excited the upper or lower polariton modes, and recorded the interpulse time dependence of the echo decay as a function of temperature (Figure S8, Supporting Information). Monoexponential fits of these data (Figure 4D) reveal that the phase memory time of the strongly coupled ensemble only changes slowly with temperature and still amounts to 600 ns, even at room temperature.

**Figure 5:**
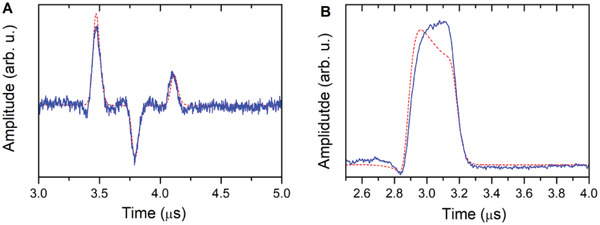
Microwave pulse storage experiments. Data were recorded on a 5.5 mg BDPA.Bz pellet at 1248.8 mT and 7 K, the microwave frequency was 35.062 GHz (upper polariton mode). Red dotted lines are simulations (see text). A) Multiple echo (in‐phase component) detected at 7 K after a pulse sequence consisting of three 30 ns pulses with a pulse power of 5 mW and an interpulse delay of 400 ns, followed after 1.4 µs by a 25 ns, 5W pulse. The phase of the first two (left) or second (right) pulses was inverted. B) Echo detected after a pulse sequence consisting of a 300 ns, 5 mW pulse followed after 1.4 µs by a 25 ns, 5W retrieval pulse.

Because coherence on the polariton modes is only generated by the pulse edges, that is, a small fraction of the actual pulse, these measurements can also be seen as the storage of weak microwave excitations into an inhomogeneously broadened system. In fact, the concept of storing information in an inhomogeneously broadened spin system dates back to 1955.^[^ ^66^, ^67^ ^]^ In this scheme, a number of electromagnetic pulses generate coherences in the ensemble. These pulses must be weak so that subsequent pulses do not change the stored information. This information quickly dephases due to the inhomogeneous distribution of Larmor frequencies, but can be recalled by a strong pulse in a second step. With the advent of cavity QED, the idea has resurfaced in recent years, where the aim is to eventually store single photons.^[^ ^32^, ^33^, ^46^, ^51^ ^]^ In the following, we explore the possibility to store microwave pulses in inhomogeneously broadened spin ensembles in more detail. To this end, we employed a hybrid system of a 5.5 mg BDPA pellet mounted in a copper FPR tuned to ω_c_/2π  =  35.000 GHz (*T*  =  7 K, Ω_eff_/2π  = 62 MHz), with an applied field strength such that that ω_s_  =  ω_c_. First, three weak (5 mW) 30 ns microwave pulses, spaced by 290 ns were applied. Their frequency was set to coincide with the upper polariton mode (35.062 GHz). The phase of the second pulse was inverted compared to that of the other two. These pulses are too weak to create measurable spin echos by themselves. After a waiting time of τ  =  1.4 µs, a strong (5 W) pulse of 22.5 ns duration was employed. As a result, at a time of 2τ  =  2.8 µs, a series of echoes was observed (**Figure** **5**A, Figure S9, Supporting Information), that correspond to the stored pulses in reverse order. In contrast to the previously discussed results, these echoes are not modulated, because the polariton mode (i.e., the eigenstate) is excited directly. Importantly, the phases of these retrieved echoes are the same as of the pulses that were stored. These results demonstrate that information in the form of microwave pulses can be successfully stored in the ensemble‐resonator system. We note furthermore, that the phase information is faithfully reproduced by the simulation. It can be seen that the echo amplitude decreases monotonously, which is due to decoherence, the characteristic time of which is the same as found above (γ_M_/2π  =  0.114 MHz). Unfortunately, we could not explore the maximum number of pulses that can be stored any further, because with four pulses, the duty cycle of our microwave amplifier is already at its limit. We repeated the microwave pulse storage experiments at different temperatures (Figure S10, Supporting Information), which revealed that even at room temperature, microwave pulse storage and retrieval is achieved. To assess the efficiency of microwave pulse storage, we carried out a further experiment, where we applied a single 300 ns (5 mW) pulse followed by a 5 W retrieval pulse. Again the simulation satisfactorily reproduced the observed echo (Figure 5B). We attribute the imperfect reproduction of the top of the pulse to the fact that our pulses are not perfectly rectangular, due to the bandwidth of the microwave amplifier. The integral of the echo signal is a measure of the signal energy. Using a calibration of the equipment by means of a fully reflected 5 µW pulse, we determined a pulse storage efficiency of η  =  *E*_r_/*E*_i_  = 2(1) × 10^–5^ at 10 K and 4(2) × 10^–6^ at 291 K (**Table** **1**, Figure S11, Supporting Information). This is many orders of magnitude better than the storage efficiency of η  =  1 × 10^−10^ reported at room temperature for a system in the weak coupling regime.^[^ ^33^, ^68^ ^]^ An comparable efficiency of 4.5 × 10^−5^ was achieved, but only at 2 K.^[^ ^46^ ^]^ Only by decreasing the thermal energy by an order of magnitude, better storage efficiencies of η  =  3 × 10^−3^ are possible.^[^ ^68^, ^69^ ^]^ Because the collective coupling strength increases with the number of spins in the samples, microwave quantum memories based in spin ensembles have macroscopic dimensions. Thus spin density is an important design criterion. Here magnetically dense organic radicals such as BDPA (1.5 × 10^18^ mm^−3^) clearly outperform NV^−^ centers (10^15^ mm^−3 [^ ^70^ ^]^), P:Si (10^14^ mm^−3 [^ ^45^ ^]^), and even other molecular solids such as VO(TPP):TiO(TPP) (2.3 × 10^16^ mm^−3 [^ ^46^ ^]^). The storage time, which is essentially given by the coherence time of the strongly coupled ensemble (Table 1) is a further important performance indicator. For more meaningful comparison to other material platforms, we have divided the storage time by the lengths of the microwave pulses employed giving the storage bandwidth, which is a measure of how many pulses can be stored. Here it can be seen that especially P:Si performs very well, due to its extraordinarily long coherence time at low temperatures. Although their envisioned applications differ from those of microwave quantum memories, the comparison to optical quantum memories is instructive (Table 1). Here we have considered two examples, namely one based on the solid state material Pr^3+^:Y_2_SiO_5_ and one based on cesium atomic gas. Retrieval efficiencies are orders of magnitude higher in these systems. On the other hand, their storage times and storage bandwidths are not too dissimilar to the BDPA/FPR system we present here, especially when considering that in the latter pulse lengths can easily be shortened by an order of magnitude. Furthermore, we note that we have carried out measurements on different BDPA samples and with different FPRs and have always obtained comparable results, underlining the robustness of the platform. One of the next steps would be now to assess the retrieval fidelity that is the overlap between the stored and retrieved photon (superposition) state.

**Table 1:** Memory storage efficiencies and temperatures compared to other spin‐based and optical quantum memories

System	Strong coupling?	Storage effciency	Storage time	Storage bandwidth	Storage temp.	Ref.
Microwaves						
^31^P:^nat^Si/LER^a)^/4.8 GHz	Y	n.d.	2.37 ms	2000	50 mK	^[^ ^45^ ^]^
NV diamond/LER/2.88 GHz	Y	3 × 10^−3^	84 µs	80	100 mK	^[^ ^69^ ^]^
VO(TPP):Ti(TPP)^b)^/CPR^c)^/6.58 GHz	Y	4.5 × 10^−5^	0.97 µs	10	2 K	^[^ ^46^ ^]^
^31^P:^28^Si/9.8 GHz	N	n.d.	450 µs	100 000	9 K	^[^ ^33^ ^]^
BDPA/3D FPR^d)^/35 GHz	Y	2(1) × 10^−5^	1.2 µs	40	10 K	^e)^
BDPA/3D FPR/35 GHz	Y	4(2) × 10^−6^	0.6 µs	20	291 K	^e)^
^14^N@C_60_	N	10^−10^	80 µs	4000	r.t.^f)^	^[^ ^33^ ^]^
Optical Photons						
Pr^3+^:Y_2_SiO_5_	N	0.69	3 µs	4	3 K	^[^ ^71^ ^]^
cesium	N	0.1	1.4 µs	700	330 K	^[^ ^72^ ^]^

^a)^ LER = lumped rlement resonator; nat = natural abundance, i.e., not isotropically purified

^b)^ TPP = tetraphenyl porphyrin

^c)^ CPR = coplanar resonator

^d)^ FPR = Fabry–Pérot resonator

^e)^ this work.

^f)^ r.t. = room temperature.

## 4 Conclusion

We have shown that very high cooperativities can be obtained when using molecular spin ensembles and 3D microwave resonators. This is because the microwave mode volume is larger than in 2D‐resonators allowing for using larger numbers of spins. We directly observed vacuum Rabi oscillations in time‐domain investigations. Here the high degree of microwave magnetic field homogeneity of 3D resonators compared to 2D resonators was of benefit, and no distribution of Rabi frequencies needed to be taken into account. Furthermore, we found unexpectedly long coherence times when using conventional Hahn echo pulse sequences. Finally, we demonstrated the feasibility of storing microwave pulses, including phase information into the spin‐ensembles, which is a prerequisite for using such hybrid systems as quantum memories. Time‐domain experiments have been reported previously, but only at (sub)Kelvin temperatures. Here we have shown the possibility of performing such measurements up to room temperature, greatly increasing the potential of magnetically dense organic radicals in hybrid cavity–spin‐ensemble quantum systems for application as robust quantum memories.

## 5 Experimental Section

The benzene complex of α,γ‐bisdiphenylene‐β‐phenylallyl (≡ BDPA·Bz) was acquired commercially. Magnetic measurements were carried out on a Quantum Design MPMS3 SQUID magnetometer. The data were corrected for diamagnetic contributions using Pascal's constants. All strong coupling experiments were carried out employing a home‐built Fabry–Pérot resonator (FPR) made of copper,^[^ ^47^ ^]^ inserted into an Oxford Instruments CF935 continuous flow helium cryostat. Samples were pressed into 5 mm pellets. Magnetic fields were applied with a Varian V‐3800 electromagnet, equipped with an Elektro‐Automatik EA‐PS 9200‐140 power supply. For all CW measurements an Anritsu MS46322B vector network analyzer was used, which was connected to the resonator via a WR28‐2.92mm waveguide‐coax transition. The VNA was calibrated up to the coax connection for a measurement range of 34.5–35.5 GHz using a SOLT calibration kit. The probe power was set to −20 dBm. At this probe power ≈10^9^ photons are inserted into the cavity, that is, far fewer than the number of spins, and probing does not influence the spin state population. Pulsed measurements were performed using a home‐built pulsed *Q*‐band (35 GHz) spectrometer, which is based on a homodyne bridge, in which the reflected microwave signal is mixed in a balance mixer with the reference arm signal and amplified by a video amplifier.^[^ ^73^ ^]^ Microwave simulations were carried using CST Microwave Studio. The continuous wavelet transforms were calculated using Matlab's Wavelet toolbox.

## Conflict of Interest

The authors declare no conflict of interest.

## Supporting information

Supporting Information

## Data Availability

The data that support the findings of this study are available from the corresponding author upon reasonable request.
