# Supplementary material for: Room‐Temperature Quantum Memories Based on Molecular Electron Spin Ensembles
Source: Adv Mater. 2021 Jun 9;33(30):2101673. doi: 10.1002/adma.202101673 (PMC11469281; doi:10.1002/adma.202101673)
Supplement: Supplementary file 1 — Supporting Information [file ADMA-33-2101673-s001.pdf]

© 2021 Wiley-VCH GmbH

ADVANCED MATERIALS

Supporting Information

for *Adv. Mater.,* DOI: 10.1002/adma.202101673

Room-Temperature Quantum Memories Based on Molecular Electron Spin Ensembles

*Samuel Lenz, Dennis König, David Hunger, and Jorisvan Slageren**

Supporting Information

Room-Temperature Quantum Memories Based on Molecular Electron Spin Ensembles

*Samuel Lenz, Dennis König, David Hunger, Joris van Slageren**

Contents

1. BDPA Structure 27
2. CW measurements 28
3. Estimate of single spin-photon coupling strength. 28
4. BDPA exchange coupling. 29
5. Effects of Inhomogeneous broadening 29
6. Time-frequency analysis of a rectangular pulse 30
7. Microwave Frequency Dependence of Echo Modulation 31
8. Temperature Dependence of the Microwave Echo Decay. 33
9. Microwave Pulse Storage 36
References 38

1. BDPA Structure

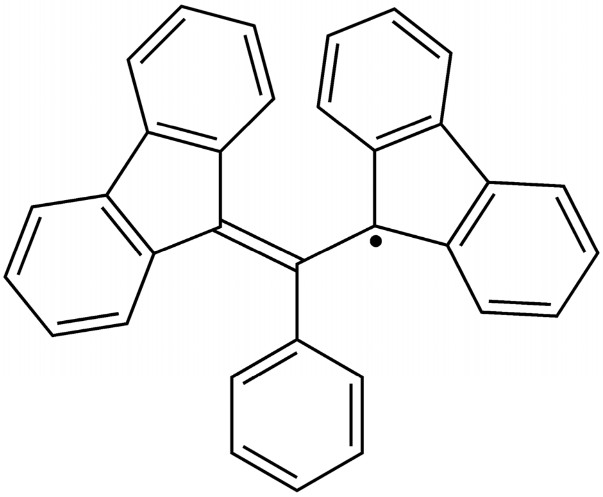

Figure S1. Molecular structure of α,γ-bisdiphenylene-β-phenylallyl ≡ BDPA.

2. CW measurements

Table S1. Measured dissipation rates, collective coupling constant and cooperativity of a 9.4 mg BDPA·Bz sample inside the copper Fabry-Pérot resonator at different temperatures.

T **(K)**	γ **/ 2π (MHz)**	κ_i **/ 2π (MHz)**	κ_e **/ 2π (MHz)**	Ω_{eff}**/ 2π (MHz)**	***C***
7	1.5(5)	2.0(5)	13.0(5)	80(3)	290(90)
9	1.5(5)	2.0(5)	13.0(5)	78(3)	270(90)
1	1.5(5)	2.0(5)	13.0(5)	73(3)	240(80)
13	1.5(5)	2.0(5)	13.0(5)	71(3)	220(70)
15	1.5(5)	2.0(5)	13.0(5)	68(2)	200(70)
20	1.5(5)	2.0(5)	13.0(5)	62(2)	170(60)
25	1.5(5)	2.0(5)	13.0(5)	58(2)	150(50)
30	1.5(5)	2.0(5)	13.0(5)	53(2)	120(40)
50	1.5(5)	2.0(5)	13.0(5)	41(2)	70(20)
70	1.5(5)	2.5(5)	13.0(5)	36(2)	60(20)
90	1.5(5)	2.5(5)	13.0(5)	31(2)	40(10)
100	1.5(5)	2.5(5)	13.0(5)	31(2)	40(10)
120	1.5(5)	2.5(5)	13.0(5)	29(2)	40(10)
180	1.5(5)	3.0(5)	13.0(5)	24(2)	25(10)
200	1.5(5)	3.5(5)	13.0(5)	23(2)	21(7)
240	1.5(5)	4.5(5)	13.0(5)	20(2)	15(5)
291	1.5(5)	5.5(5)	13.0(5)	19(2)	13(5)

3. Estimate of single spin-photon coupling strength.

The expression for the single spin-photon coupling strength depends on the magnetic field generated by a single photon in the cavity as $g_s = |B_{\text{photon}}|(g\mu_B/2\hbar) = \sqrt{\hbar\omega\mu_0/2V_{\text{mode}}}\,(g\mu_B/2\hbar)$. The simulated single photon field $B_{\text{photon,sim}}$ can be obtained from microwave simulations by $|B_{\text{photon, sim}}| = |B_{\text{sim}}|/\sqrt{N_{\text{p}}}$, with Np the number of photons in the cavity given by $N_p = E_c/\hbar\omega_c$ with E_c the energy stored in the cavity and ω_c the photon frequency. Carrying out such simulations for the different cavity modes resulted in single spin-photon coupling parameters between 72 and 139 mHz (Table S2). The clear proportionality between collective coupling strength and simulated single-spin coupling strengths found for when using four different cavity modes (Table S2, Figure S2) supports the validity of this approach.

Table S2. Measured dissipation rates, collective coupling constant and cooperativity of a 18 mg BDPA·Bz sample inside the copper Fabry-Pérot resonator.

Mode	γ **/ 2π (MHz)**	κ_i **/ 2π (MHz)**	κ_e **/ 2π (MHz)**	Ω_{eff}**/ 2π (MHz)**	$g_{s,sim}$**/2π (Hz)**	***C***
1	1.5(5)	8.0(5)	13.0(5)	28.0(5)	0.139	25(10)
2	1.5(5)	6.5(5)	7.0(5)	19.0(5)	0.099	21(7)
3	1.5(5)	4.0(5)	7.0(5)	12.0(5)	0.072	15(5)
4	1.5(5)	4.0(5)	4.0(5)	13.0(5)	0.076	13(5)

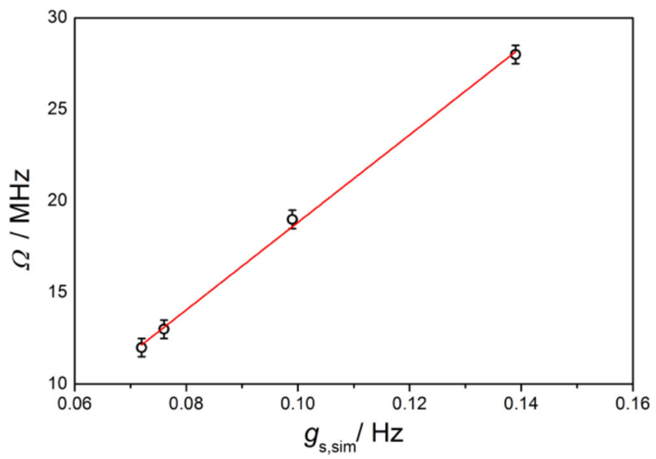

Figure S2. Measured collective coupling Ω_{eff} (black symbols) of a 18 mg BDPA·Bz sample for the first four modes occurring in the Fabry-Pérot resonator versus the simulated single spin coupling of the given mode.

4. BDPA exchange coupling.

Figure S2 shows the susceptibility and susceptibility-temperature product recorded on a pressed powder sample of BDPA. The spin Hamiltonian for an infinite ($N_c \to \infty$) Heisenberg chain is given by:

$$\mathcal{H} = g\mu_B B_0 \sum_{i=1}^{N_c} \hat{S}_{z,i} - 2J \sum_{i=1}^{N_c} \hat{S}_i \cdot \hat{S}_{i+1} \qquad \text{(Eq. S6)}$$

Where the g-value is $g = 2.0027$,[1] μ_B is the Bohr magneton, B_0 the applied magnetic field and J the Heisenberg (isotropic) exchange coupling constant. Approximating the infinite chain susceptibility by the average value for $N_c = 11$ and $N_c = 12$ odd- and even-membered rings, the susceptibility was fitted, yielding an exchange coupling constant of $J/k_B = -4.9$ K. The increase in χ at the lowest temperatures was reproduced by including a small amount (4%) of uncoupled impurity in the fit.

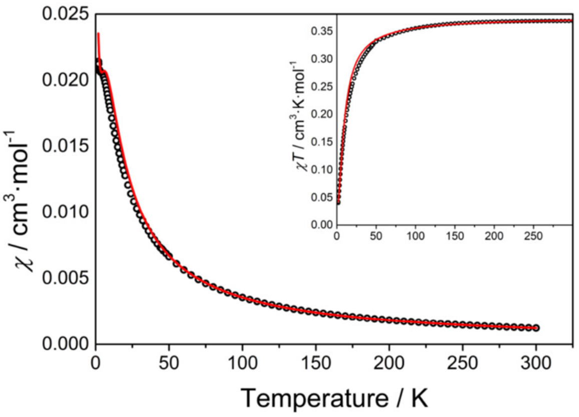

Figure S3. Magnetic susceptibility χ, measured (symbols) on a pressed powder pellet of BDPA using an applied field of 0.1 T ($T < 50$ K) and 1 T ($T > 40$ K). The inset shows the χT product. Solid lines are fits based on the spin Hamiltonian given in the text.

5. Effects of Inhomogeneous broadening

Equation S4 gives the S_{11} scattering parameter for a collection of ensembles (spin packets). Given a spectral distribution of the spin resonance frequencies $\rho(\omega_{s,j})$, the number of spins with identical properties in each spin packet N_j is given by $N_j = N_{tot}\,\rho(\omega_{s,j}) \Big/ \sum_{j=1}^{N} \rho(\omega_{s,j})$. Here we have chosen the q-Gaussian spectral distribution function:

$$\rho(\omega_{s,j}) = \left[1-(1-q)\frac{(\omega_{s,j}-\omega_{s,c})^2}{\Delta^2}\right]^{\frac{1}{1-q}} \quad ; \qquad \gamma_{inh} = 2\Delta\sqrt{\frac{2^q-2}{2q-2}} \qquad \text{(Eq. S7)}$$

This distribution becomes Gaussian for $q \rightarrow 1$, and Lorentzian for $q = 2$. In this equation $\omega_{s,j}$ is the spin resonance frequency of the jth spin packet and $\omega_{s,c} = \omega_{s,c}(B_0)$ is the spin resonance frequency at the center of the distribution. The parameter Δ is a measure of the width of the distribution and is related to the inhomogeneous FWHM line width γ_{inh} as given in the equation. Our fits yielded a q-parameter of $q = 1.1$.

6. Time-frequency analysis of a rectangular pulse

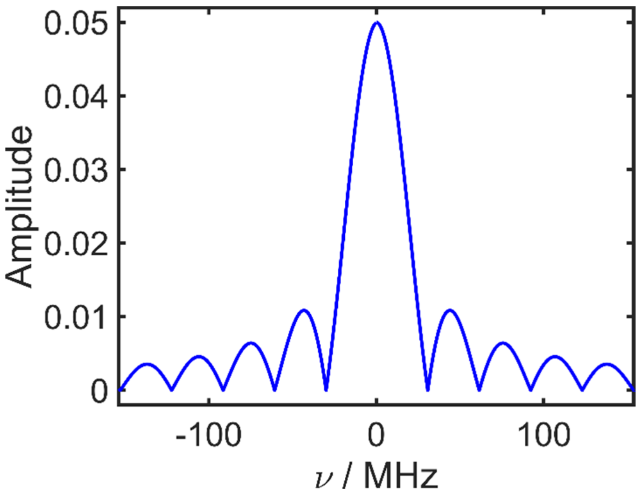

Figure S4. Fast Fourier Transform of a 65 ns rectangular pulse

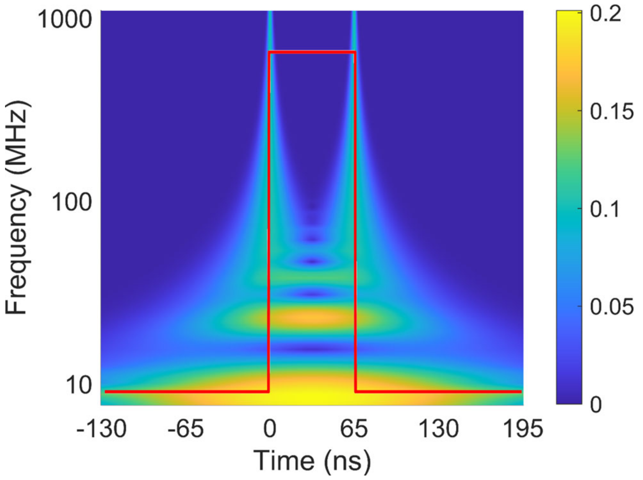

Figure S5. Continuous wavelet transform of a 65 ns rectangular pulse. The red line indicates schematically the rectangular pulse.

7. Microwave Frequency Dependence of Echo Modulation

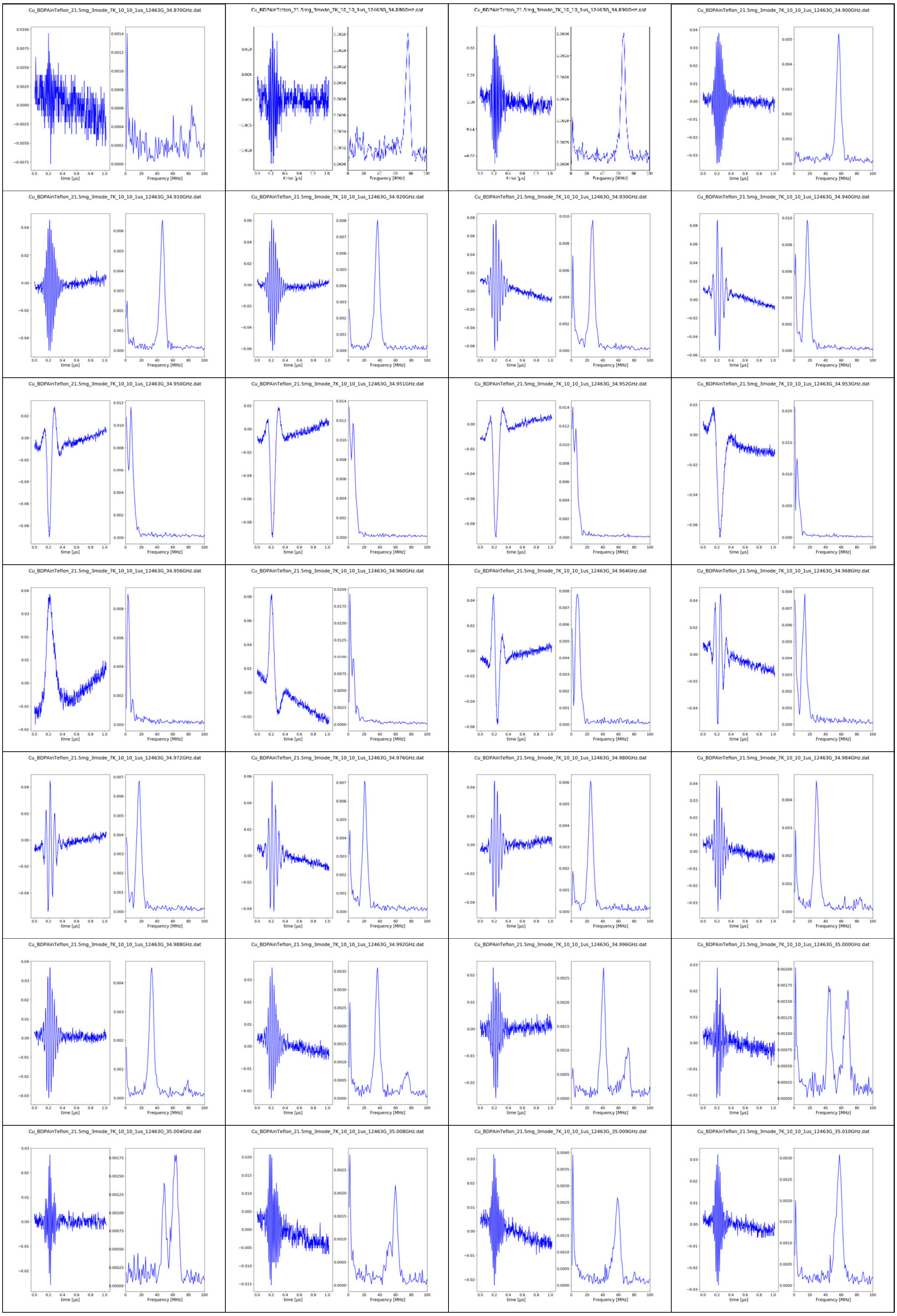

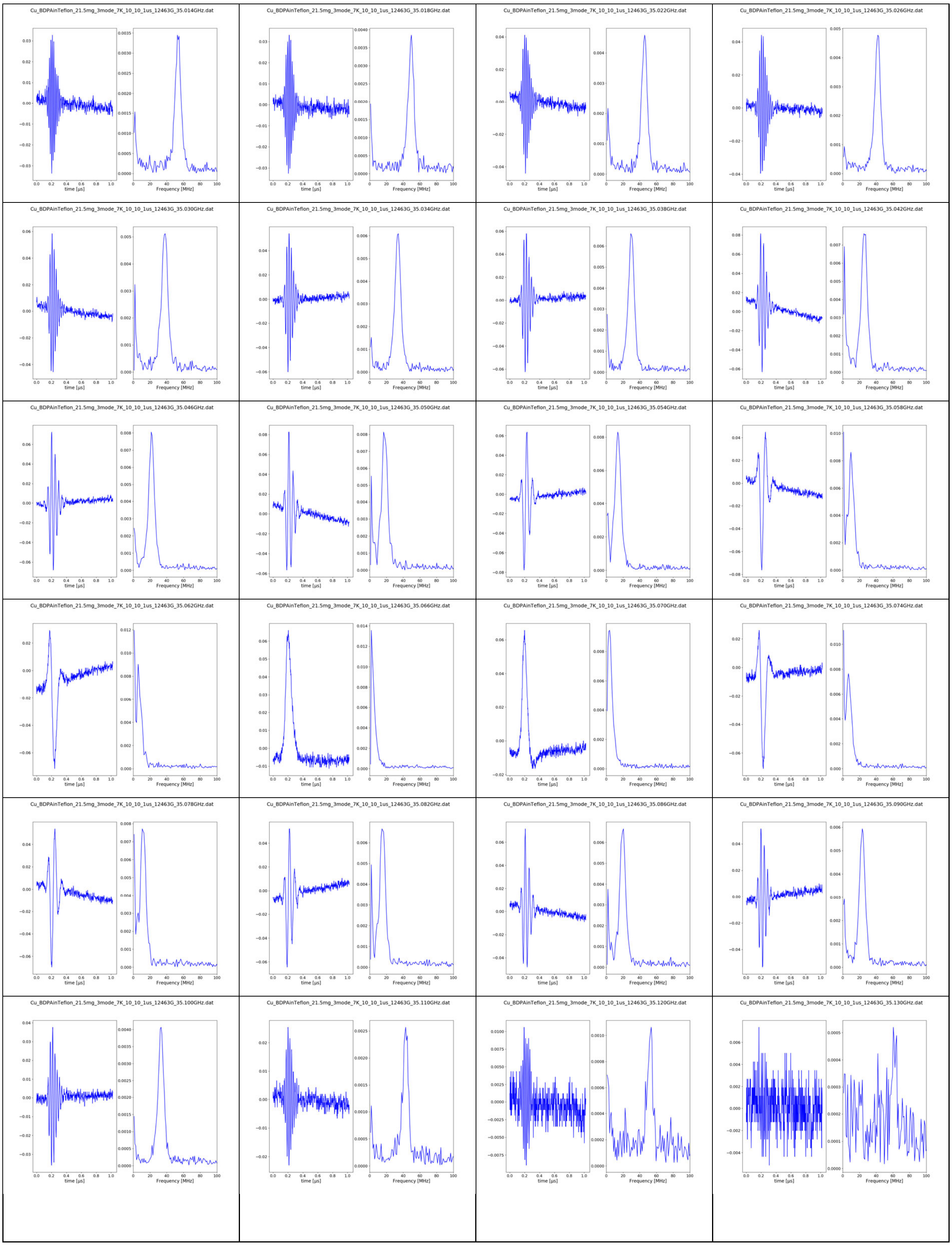

Figure S6. Microwave echoes (left hand side of each panel) recorded on a 21.5 mg BDPA sample at 7 K after two 10 ns, 5 W microwave pulsed with a 1 µs interpulse delay, using different microwave frequencies. The right hand side of each panel is the FFT of the echo signal.

8. Temperature Dependence of the Microwave Echo Decay.

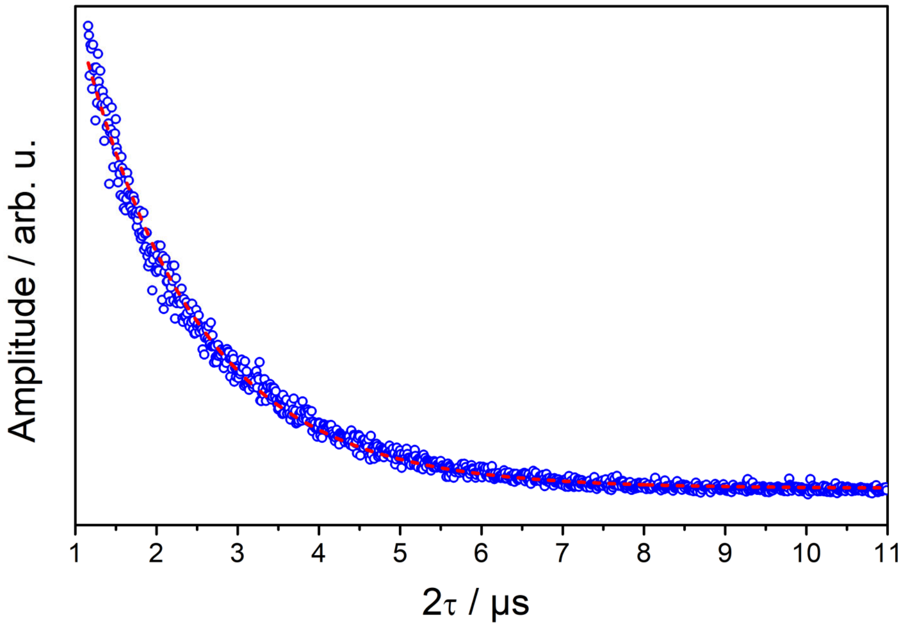

Figure S7. Peak to peak amplitude of the echo signal (blue symbols) as a function of twice the interpulse delay time τ, recorded after applying two 25 ns, 5 W pulses at 7K and $\omega_p = \omega_c = \omega_s = 2\pi\cdot 35.000$ GHz (zero detuning).

***T*/K**	**Lower Polariton Mode**	**Upper Polariton Mode**
10 K	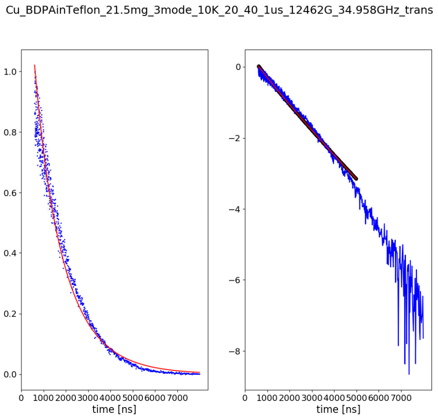	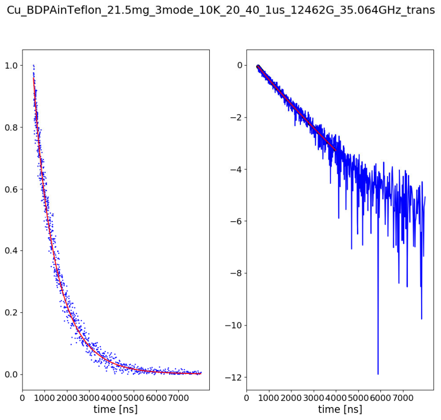
20 K	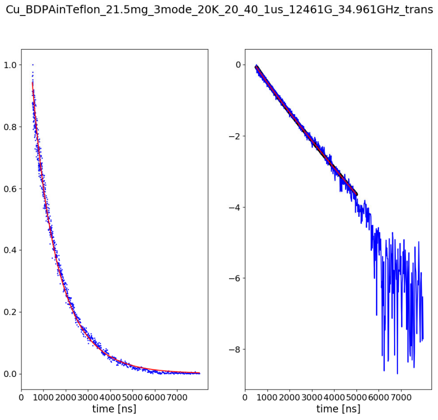	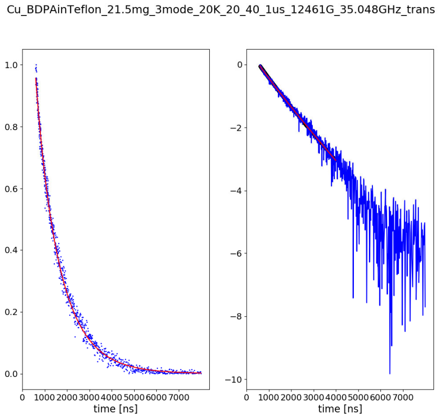

40 K

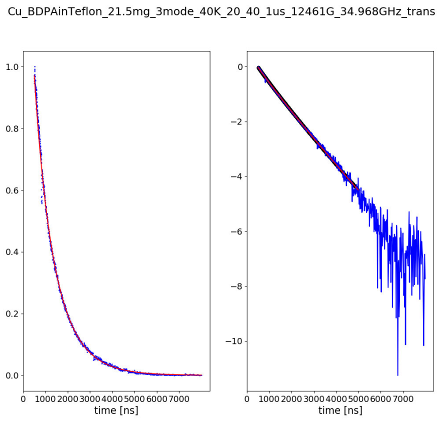

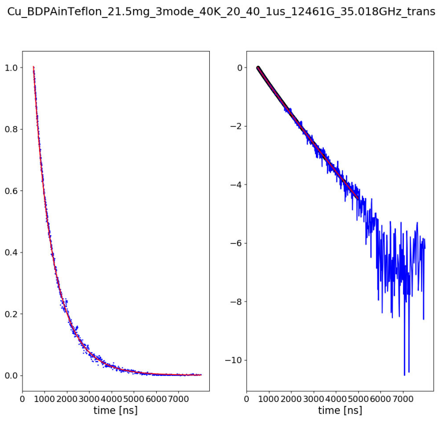

60 K

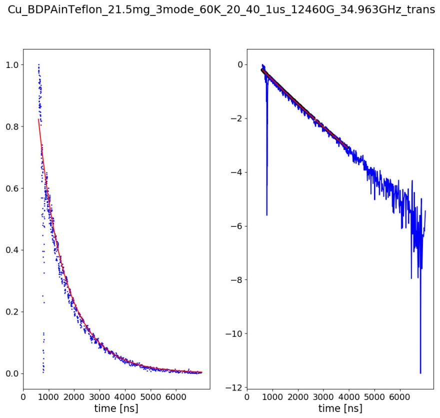

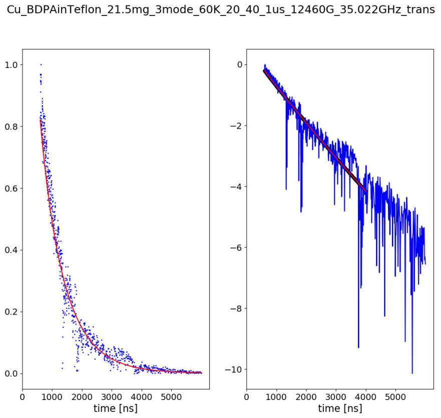

100 K

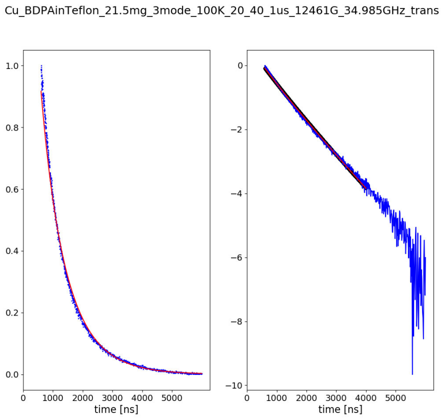

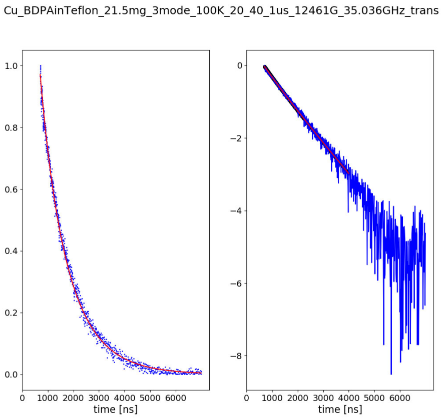

150

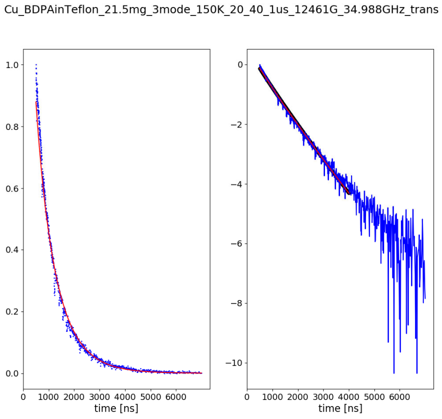

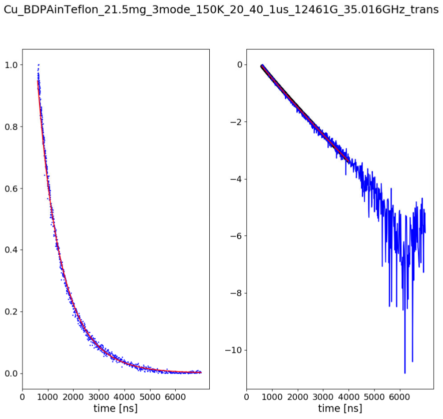

291

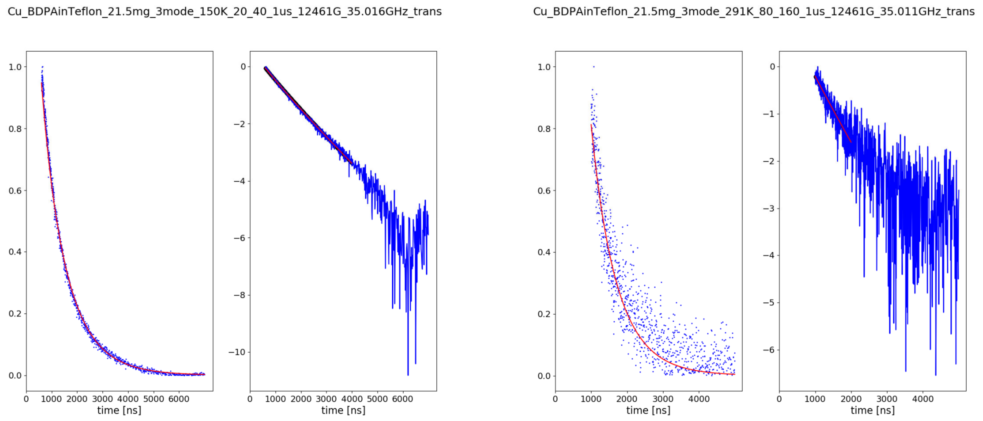

Figure S8. Microwave echo intensity recorded on a 21.5 mg powder sample of BDPA after two 5 W pulses with 20 ns and 40 ns duration as a function of twice the interpulse delay at different temperatures as indicated.

Table S3. Phase Memory Times obtained by fitting the echo decay curves of Figure S7 to monoexponential decay functions.

T / K	T_M Lower Polariton Mode / ns	T_M Upper Polariton Mode / ns
10	1144	913
20	1041	952
40	831	834
60	968	721
100	937	761
150	858	707
291	405	622

9. Microwave Pulse Storage

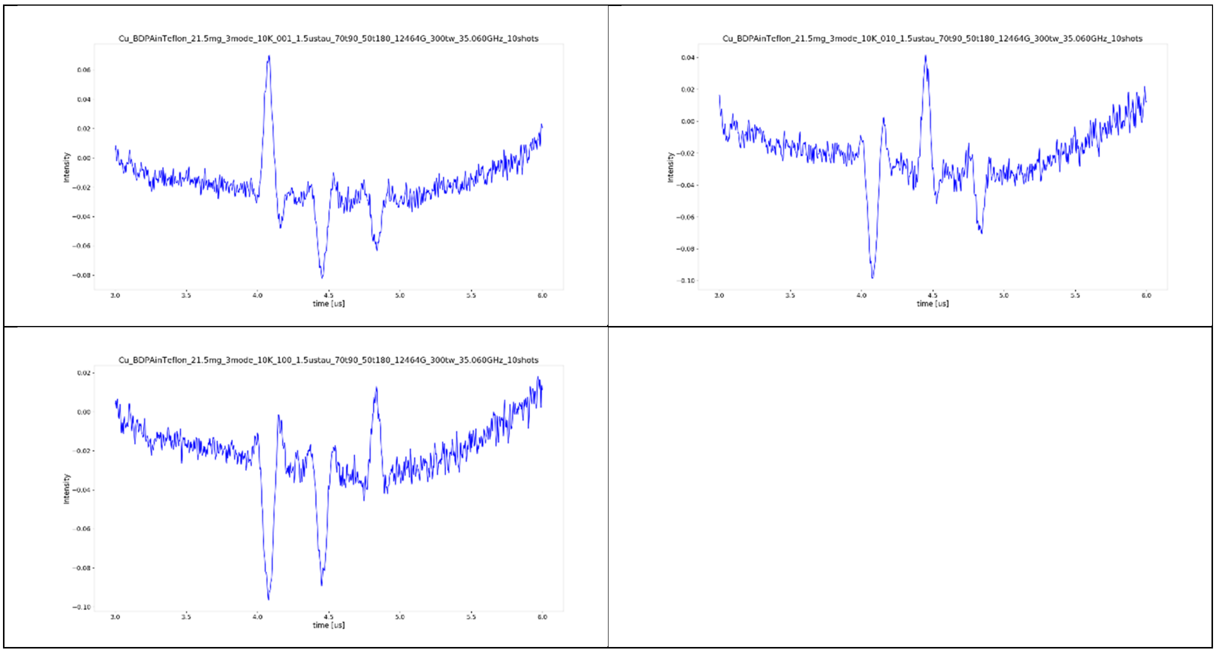

Figure S9. Multiple echo (in-phase component) detected at 10 K after a pulse sequence consisting of three 70 ns pulses with a pulse power of 5 mW and an interpulse delay of 400 ns, followed after 1.5 µs by a 50 ns, 5W pulse. The different panels show different phases of the stored microwave pulses.

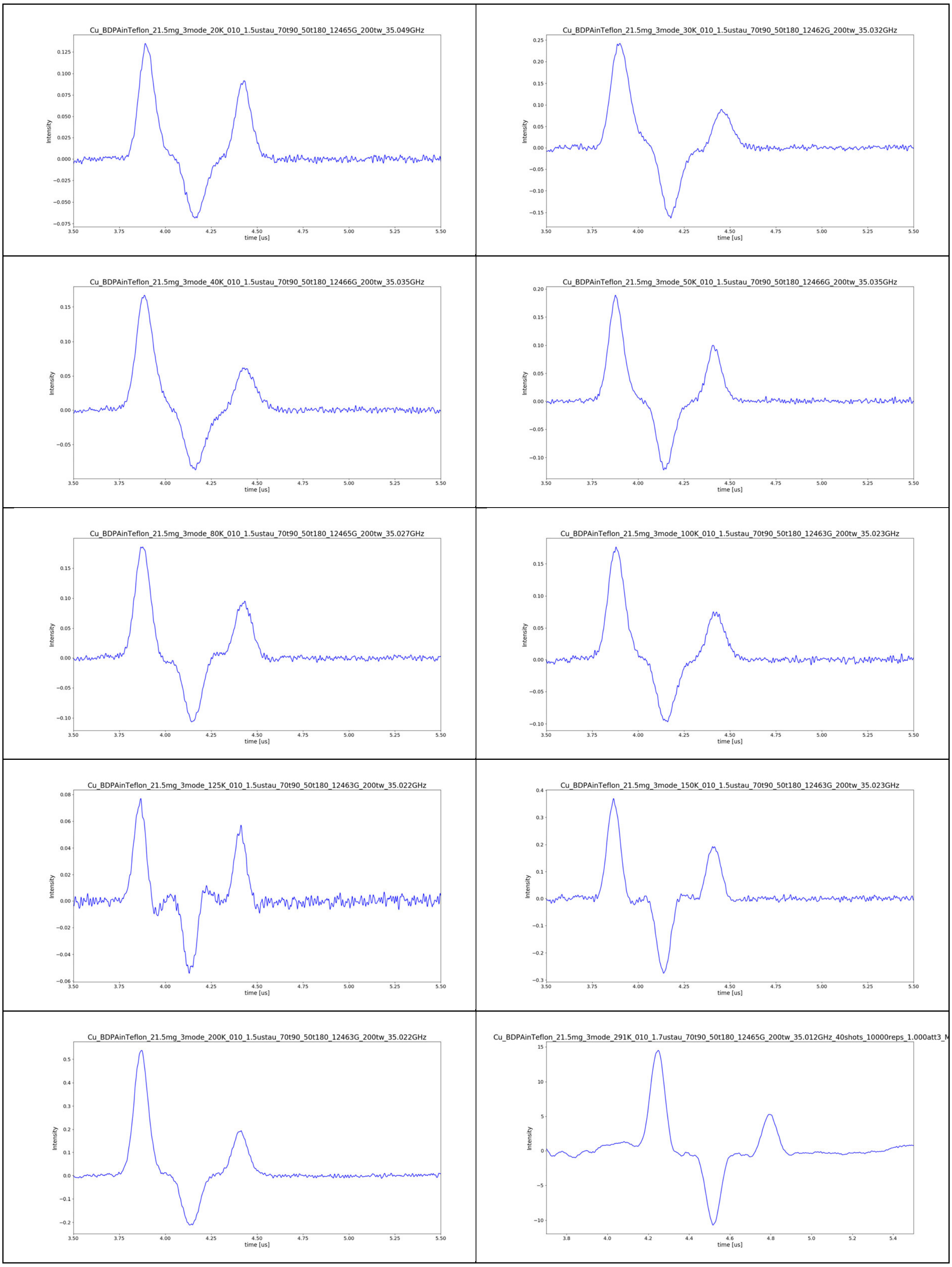

Figure S10. Multiple echo (in-phase component) detected at different temperatures after a pulse sequence consisting of three 70 ns pulses with a pulse power of 5 mW and an interpulse delay of 400 ns, followed after 1.5 μs by a 50 ns, 5W pulse.

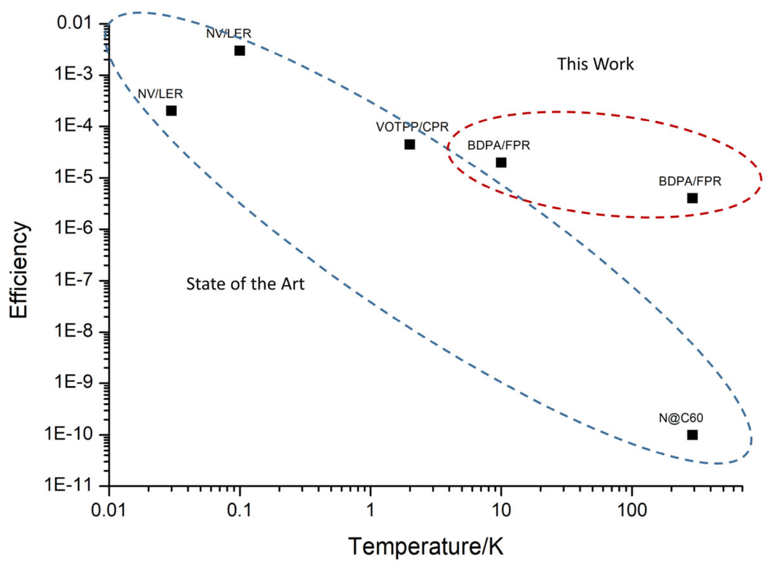

Figure S11. Graphical overview over state of the art in terms of microwave pulse storage efficiency and operating temperatures, see also Table 1.

References

[1] A. Abragam, B. Bleany, *Electron Paramagnetic Resonance of Transition Ions*, Dover Publications, Inc., New York, **1986**.
